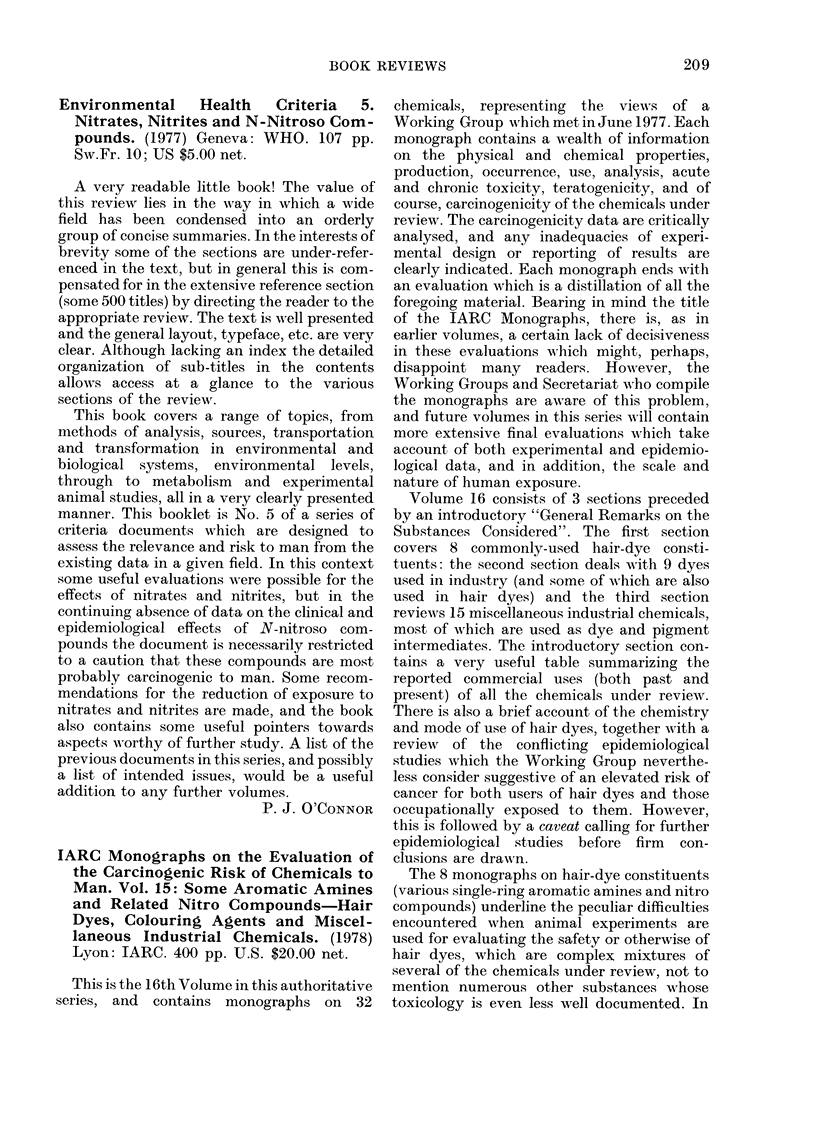# Environmental Health Criteria 5. Nitrates, Nitrites and N-Nitroso Compounds

**Published:** 1979-02

**Authors:** P. J. O'Connor


**Environmental Health Criteria 5. Nitrates, Nitrites and N-Nitroso Compounds.** (1977) Geneva: WHO. 107 pp. Sw.Fr. 10; US $5.00 net.

A very readable little book! The value of this review lies in the way in which a wide field has been condensed into an orderly group of concise summaries. In the interests of brevity some of the sections are under-referenced in the text, but in general this is compensated for in the extensive reference section (some 500 titles) by directing the reader to the appropriate review. The text is well presented and the general layout, typeface, etc. are very clear. Although lacking an index the detailed organization of sub-titles in the contents allows access at a glance to the various sections of the review.

This book covers a range of topics, from methods of analysis, sources, transportation and transformation in environmental and biological systems, environmental levels, through to metabolism and experimental animal studies, all in a very clearly presented manner. This booklet is No. 5 of a series of criteria documents which are designed to assess the relevance and risk to man from the existing data in a given field. In this context some useful evaluations were possible for the effects of nitrates and nitrites, but in the continuing absence of data on the clinical and epidemiological effects of *N*-nitroso compounds the document is necessarily restricted to a caution that these compounds are most probably carcinogenic to man. Some recommendations for the reduction of exposure to nitrates and nitrites are made, and the book also contains some useful pointers towards aspects worthy of further study. A list of the previous documents in this series, and possibly a list of intended issues, would be a useful addition to any further volumes.

P. J. O'Connor